# Telomeres in Interstitial Lung Disease

**DOI:** 10.3390/jcm10071384

**Published:** 2021-03-30

**Authors:** Carmel J. W. Stock, Elisabetta A. Renzoni

**Affiliations:** Interstitial Lung Disease Unit, Royal Brompton and Harefield Clinical Group, Guy’s and St Thomas’ NHS Foundation Trust/National Heart and Lung Institute, Imperial College London, Sydney Street, London SW3 6NP, UK; E.Renzoni@rbht.nhs.uk

**Keywords:** telomeres, telomerase, telomere related genes, ILD, IPF, genetics

## Abstract

Interstitial lung diseases (ILD) encompass a group of conditions involving fibrosis and/or inflammation of the pulmonary parenchyma. Telomeres are repetitive DNA sequences at chromosome ends which protect against genome instability. At each cell division, telomeres shorten, but the telomerase complex partially counteracts progressive loss of telomeres by catalysing the synthesis of telomeric repeats. Once critical telomere shortening is reached, cell cycle arrest or apoptosis are triggered. Telomeres progressively shorten with age. A number of rare genetic mutations have been identified in genes encoding for components of the telomerase complex, including telomerase reverse transcriptase (*TERT*) and telomerase RNA component (*TERC*), in familial and, less frequently, in sporadic fibrotic ILDs. Defects in telomerase result in extremely short telomeres. More rapidly progressive disease is observed in fibrotic ILD patients with telomere gene mutations, regardless of underlying diagnosis. Associations with common single nucleotide polymorphisms in telomere related genes have also been demonstrated for various ILDs. Shorter peripheral blood telomere lengths compared to age-matched healthy individuals are found in a proportion of patients with fibrotic ILDs, and in idiopathic pulmonary fibrosis (IPF) and fibrotic hypersensitivity pneumonitis (HP) have been linked to worse survival, independently of disease severity. Greater susceptibility to immunosuppressant-induced side effects in patients with short telomeres has been described in patients with IPF and with fibrotic HP. Here, we discuss recent evidence for the involvement of telomere length and genetic variations in the development, progression, and treatment of fibrotic ILDs.

## 1. Interstitial Lung Disease

Interstitial lung diseases (ILD) comprise a diverse group of entities involving varying admixtures of fibrosis and inflammation in the pulmonary parenchyma. Major entities include the idiopathic interstitial pneumonias (IIPs) (e.g., idiopathic pulmonary fibrosis (IPF) characterised by a usual interstitial pneumonia (UIP), as the most frequent, followed by fibrotic non-specific interstitial pneumonia (NSIP)), those related to environmental exposure (e.g., hypersensitivity pneumonitis (HP) and pneumoconioses), those secondary to connective tissue disease (CTD) (most frequently scleroderma-ILD (SSc-ILD), rheumatoid arthritis-ILD (RA-ILD), and myositis-associated ILD), pulmonary sarcoidosis, and smoking-related ILDs. More newly described entities include idiopathic pleuroparenchymal fibrosis (iPPFE), acute fibrinous and organising pneumonia (AFOP), familial ILD, subclinical ILD, and interstitial lung abnormalities (ILAs), while it is recognised that unclassifiable ILD is encountered in a substantial minority of cases [1,2]. Of note, in the context of familial ILD, although a UIP pattern represents the most common pathological pattern, there is a high proportion of families with the same gene mutation with different morphological patterns between affected family members [3]. Furthermore, in familial ILD, atypical CT patterns (inconsistent with either UIP or fibrotic NSIP) are often seen [3]. Therefore, throughout this review, we have not attempted to rigidly divide telomere findings according to a diagnosis of IPF or non IPF, although have attempted to clarify where genetic association were identified with ILDs with known causes or associations, including connective tissue disease or hypersensitivity pneumonitis.

## 2. Telomeres

In mammals, telomeres are TTAGGG tandem repeat DNA sequences at the end of chromosome arms which maintain genome stability by shielding chromosome ends from shortening during replication. Telomeres shorten with each cell division. When telomere length becomes critically short, the cell enters senescence or apoptosis [4]. Telomere length progressively shortens with age [5], and telomere shortening is suggested as one of the key mechanisms underlying the aging processes. A large number of genes are involved in the maintenance and function of telomeres, collectively known as the telosome, encoding for the telomerase complex. This multi-subunit enzyme extends telomere length at each cell division, partially counteracting telomere shortening.

## 3. Telomeres and Interstitial Lung Diseases

The incidence of IPF exponentially increases with age, and can be viewed, at least in part, as an aberration of the normal aging process of the lung [6]. Other fibrotic ILDs also increase in frequency with age, including the other fibrotic IIPs, fibrotic HP, and RA-ILD [7,8,9]. Interstitial lung abnormalities (ILAs) are defined as incidentally discovered interstitial changes seen on CT potentially compatible with an ILD [10]. ILAs increase in frequency in older individuals (>60 years old), with a frequency of 4–9% in smokers and 2–7% in non-smokers. ILAs are associated with increased mortality and at 5 years, progress in approximately 40% of individuals [10]. The relationship between ILAs and ILD can be viewed as similar to that between ‘mild cognitive impairment’ and dementia, with a proportion of patients with ILA going on to develop frank fibrotic ILD, likely based on a combination of genetic and environmental factors.

The first association between genetically determined telomere abnormalities and lung fibrosis was observed for the telomeropathy dyskeratosis congenital (DC), an entity characterised by skin abnormalities, bone marrow failure, and pulmonary fibrosis, observed in 19% of patients [11]. The first DC gene variant to be identified was the X-linked gene dyskerin pseudouridine synthase 1 (*DKC1*), important in telomerase stabilisation and maintenance [12]. Mutations in other telomere related genes (TRGs) have also been linked to DC, and, crucially, have also been identified in familial and sporadic IIPs [13,14,15,16]. TRGs encode components of the telomerase complex, and include the telomerase reverse transcriptase (*TERT*) catalytic subunit and the telomerase RNA component (*TERC*), poly(A)-specific ribonuclease (*PARN*)*,* involved in telomere maturation, regulator of telomere elongation helicase 1 (*RTEL1*), involved in DNA helicase activity, and TERF1 interacting nuclear factor 2 (*TINF2*), involved in shelterin function [17]. Patients with functional TRG mutations are expected to have shortened age-adjusted telomere length (TL) [18]. The telomerase complex genes have been extensively reviewed elsewhere [19,20].

The pathogenic mechanisms underlying the link between telomere abnormalities and lung fibrosis are not yet fully understood [21]. When telomeres become critically short they activate a DNA damage response leading to apoptosis, senescence or a combined phenotype [22]. Senescent cells have an altered gene expression profile, and secrete a range of cytokines and growth factors (senescence associated secretory phenotype or SASP) which is likely to play a direct role in pathogenesis. Loss of regenerative potential of alveolar type II epithelial cells (AT2) cells following injury has been postulated to underlie telomeropathy-associated lung fibrosis, with concomitant excessive proliferation of airway cells displaying abnormal phenotypes [23]. However, experimental evidence for the role of pathological telomere function in the pathogenesis of inflammation and fibrosis remains scarce.

Shorter TLs are described in AT2 cells of IPF lungs compared to controls, although interestingly this was observed regardless of the presence of mutations [24]. McDonough et al. observed that explanted IPF lungs were characterised by shorter TL than control lungs. Shorter TL was associated with total collagen but not with structural changes in disease severity or with microscopic honeycombing [25]. In patients with IPF, the lung had the shortest TL compared to kidney, thyroid, liver, and bladder, with no correlation between lung TL and age, and no difference between TL in diagnostic biopsies and explanted lungs with end-stage disease, with a median of 45 months difference, showing no variation in TL over disease duration [26]. There was also no difference in TL between apical and basal lung tissue, but AT2 cell TL was longer in non-fibrotic areas compared to fibrotic areas of the same biopsy [27]. Little is known on the role of telomere abnormalities in the biology of lung fibroblasts, the main effector cells in lung fibrosis. Although lung fibroblast TL was not significantly different between control, IPF and SSc patients, telomerase activity appeared to be induced more frequently in IPF and in SSc-ILD lung fibroblasts, compared to control lung fibroblasts [28].

AT2 cells from mice with short telomeres demonstrated limited regenerative capacity due to proliferative arrest. Inducing telomere dysfunction in adult AT2 cells by deleting telomeric repeat binding factor 2 (*Trf2*), which plays a central role in telomere maintenance, preferentially activated a cellular senescence program and resulted in an inflammatory response with up-regulation of immune-signalling pathways. Challenging the mice with bleomycin resulted in 100% mortality [29]. Conditional deletion of telomere repeat binding factor 1 (*Trf1*), a component of the shelterin complex involved in the regulation of TL, in AT2 cells in mice resulted in increased mortality compared to controls, and a greater extent of lung remodelling and spontaneous fibrosis. Spontaneous lung fibrosis was specific to AT2 cells with an increased number of senescent cells in *Trf1* deleted mice [30]. Two mouse models of IPF have been developed; telomere deficient *Tert* knockout mice form critically short telomeres, while mice with AT2 *Trf1* deletion have telomere dysfunction in the absence of telomere shortening. Despite normal TL, the *Trf1* deleted mice showed CT findings and histopathology consistent with fibrosis, with laboured and heavy breathing and reduced survival, while the wild-type mice did not. Although the *Tert* knockout mice with short TL did not spontaneously develop lung fibrosis, treatment with low-dose bleomycin, insufficient to induce fibrosis in wild-type mice, induced lung fibrosis on both CT and histopathology [31]. Treating these *Tert* knockout mice previously treated with low-dose bleomycin, with Adeno-associated vector 9-*Tert* resulted in a reduction in the amount of fibrosis assessed by CT lung volume estimation, and improved spirometry, while worsening fibrosis and reduced lung function were observed in mice treated with empty vector. A reduction in the amount of fibrosis histologically, lower inflammatory markers and collagen deposition, decreased DNA damage, apoptosis and cell senescence were also observed, with treatment resulting in an increased number of AT2 cells [32].

## 4. Peripheral Blood Telomere Length and ILDs

### 4.1. Familial and Sporadic IIPs

Peripheral blood TL is considered a marker of biological age [33,34]. Patients with familial IIPs and mutations in *TERT* and *TERC* were the first to be shown to have short peripheral blood TL [18,35]. Subsequently, a significant proportion of patients with sporadic IIP without an identifiable TRG mutation, were also shown to have shorter blood leukocyte TL than age-matched controls, with reports ranging from 5.71% to 68.2% of patients [24,28,36,37,38,39,40]. Discrepancies in the rate of detection are possibly due to the different TL measurement techniques utilised (discussed below). In 75 asymptomatic first-degree relatives of familial pulmonary fibrosis (PF) patients, shorter TL was seen compared to healthy controls, with 36% having TL < 10th percentile for age [41].

### 4.2. Across ILDs

Snetselaar R et al. measured peripheral blood cell TL across a wide range of ILD diagnoses, and observed a significant difference in age-adjusted TL between controls and patients with sarcoidosis, HP, connective tissue disease-associated interstitial lung disease (CTD-ILD), iNSIP, smoking related-ILD, IPF and FIP. The largest difference from controls was seen in patients with IPF, the smallest in sarcoidosis. Despite a significant linear relationship with age in controls, there was no relationship between age and TL in the ILD cohorts [38]. In another study by Stuart B.D. and coauthors, patients with CTD-ILD, HP and IIP all had shorter age-adjusted TL compared to controls, while drug/radiation-induced ILD and sarcoidosis were not different compared to controls, but cohort numbers were small [36].

Uncertainty still exists as to whether shorter TLs are associated with worse survival or more rapid lung function worsening across all ILDs. In IPF, a stepwise decrease in survival according to TL quartiles was observed even after adjusting for age, sex, forced vital capacity (FVC) and diffusing capacity of the lung for carbon dioxide (DLCO) [37]. This was confirmed by Stuart et al., reporting that shorter TL was associated with poorer transplant-free survival in IPF across two independent cohorts, although this was not the case for non-IPF ILDs [36]. However, others have found associations between TL and survival in non-IPF ILDs. Despite patients with HP having longer TL than patients with IPF, in HP short TL was associated with more fibrosis on HRCT, and with shorter survival [42]. In a cohort of 219 patients with unclassifiable ILD, age adjusted TL was associated with presence of moderate to severe fibrosis, and traction bronchiectasis on HRCT, but was not associated with a UIP pattern. In unclassifiable ILD, TL was associated with transplant-free survival, even after adjusting for potential confounders, with patients with TL in the lowest quartile having transplant-free survival nearly identical to that of IPF patients [43]. On the other hand, Newton C.A. et al. observed that TL < 10th percentile was associated with transplant-free survival in IPF and interstitial pneumonitis with autoimmune features (IPAF), but not in CTD-ILD [44]. Compared to SSc-ILD and other CTD-ILD, patients with RA-ILD had the shortest age-adjusted TL. TL < 10th percentile was associated with faster decline in FVC in IPF and IPAF, with a trend to faster decline in CTD-ILD which did not reach significance following correction for multiple testing [44]. It may be that telomere abnormalities are more tightly associated with histological pattern rather than with the clinical diagnosis. In a histological analysis, Lee et al. reported that short telomeres and markers of senescence were expressed in AT2 cells of both IPF and non-IPF UIP biopsies [45]. It will be interesting to see whether these findings are indeed not found in non-UIP patterns of fibrotic ILD such as fibrotic NSIP.

## 5. Measuring Telomere Length

There are a number of different methods for measuring TL, each with their own advantages and drawbacks. Flow-fluorescence in situ hybridisation (FISH) is considered the gold standard, in light of its excellent inter- and intra-assay repeatability. However, Flow-FISH is not available in many laboratories, requires fresh blood, and is costly. Southern blot from DNA is an alternative method characterised by good reproducibility and good correlation with Flow-FISH, but is also not widely available, requires large quantities of DNA, is unsuited to high-throughput testing and is expensive. A widely available method for measuring TL, which is suitable for high-throughput testing in population based studies, is relatively low cost, and requires small amounts of DNA available from most archived samples, is quantitative PCR (qPCR). However, its accuracy is debated, with some studies showing very poor correlation with Flow-FISH [46,47], although others have reported acceptable correlation with Southern blot [40]. The relative merits of the methods used to measure TL in individual studies must therefore be taken into account when interpreting results [48,49,50].

## 6. Rare Gene Mutations and ILDs

Short telomere length can be determined by mutations in TRGs. By definition, mutations are rare genetic variations, occurring in <1% of the population. Although mutations in *TERT* and *TERC* are the most common, mutations in other TRGs have also been identified in ILDs, including *PARN*, *RTEL1* [51,52,53,54], *DKC1* [55], and *TINF2* [56]. Their prevalence across ILDs ranges depending on the study [57,58]. The rare, ILD associated TRG mutations discussed in this section are summarised in Table 1. TRG mutations have most commonly been reported in familial PF, although have been increasingly described also in sporadic disease. One study found that TRG mutations account for up to 25% of familial PF, 10% of sporadic IPF, and 10% of CTD-ILD [50]. In another study, 13% of familial and sporadic PF patients carried mutations in *TERT* or *TERC*, while none were identified in healthy controls. Overall, 25% of sporadic and 37% of familial PF cases had TL < 10th percentile of the controls, with all patients carrying mutations having TL < 10th percentile [13]. Of 1150 sporadic IPF patients, 4.2% carried functional *TERT* variants, compared to only 1.7% of non-ILD controls (including patients with age-related macular degeneration, RA and asthma) [59]. Borie and coauthors reported a prevalence of 16.8% of *TERT*/*TERC* disease-associated variants in patients with familial and sporadic pulmonary fibrosis [60], while deleterious variants in *TERT*, *RTEL1*, and *PARN* were seen in 11.3% of IPF patients, compared to only 0.3% of controls in the study by Petrovski et al. [61]. Of patients with RA-ILD, 11.9% carried mutations in TRGs, and had shorter TL compared to healthy controls [62]. In two cohorts of fibrotic HP, rare (minor allele frequency < 0.005) genetic variants in TRGs predicted to be damaging to protein function were identified in 11.1% and 8.1% of patients, respectively. TL was significantly shorter in patients carrying these variants, which were also associated with shorter transplant-free survival [63]. Of ten iPPFE patients tested for mutations in *TERT* and *TERC*, mutations in *TERT* were identified in five patients [64].

With regard to family members, telomere-related mutations exhibit incomplete penetrance, with not all individuals carrying mutations having short TL or developing ILD [58]. In families with heterozygous *TERT* coding mutations, 40% of mutation carriers had clinical PF, with mutation carriers having shorter TL than unrelated healthy controls [14], or related non-mutation carriers [24]. Armanios M.Y. et al. reported a prevalence of 8% for mutations in *TERT* or *TERC* in familial IP probands. TL was shorter in both probands and asymptomatic mutation carriers compared to relatives who did not carry mutations [18]. In families with idiopathic ILD and mutations in *TERT* and *TERC*, TL was shorter in individuals carrying mutations with clinical disease compared to asymptomatic carriers [35].

With regard to demographics and clinical characteristics, the average age of patients with rare variants in TRGs was significantly younger than in patients without mutations, and mutation carriers had a more rapid decline in FVC [59]. Lower transplant-free survival in patients carrying *TERT*/*TERC* mutations was seen compared to non-carriers [60]. In PF families with mutations in four genes linked to telomere shortening, *TERC* mutations carriers were diagnosed at a younger age, while *PARN* mutation carriers, who also had longer TL, were diagnosed at a later age, compared to the mean age of mutation carriers [15]. An earlier age of onset of PF in *TERC* mutation carriers (early 50s) compared to *TERT* and *PARN* mutation carriers (mid to late 60s) was also reported by Courtwright A.M. et al. [58]. Despite variability in age of onset, no difference in the rate of lung function decline, or time to death or transplantation, was observed between different TRGs [15,16]. Although initially identified in patients with IPF, mutations in TRGs are found in all ILDs, and the same mutation can be associated with different ILD patterns, even between members of the same family. In families with ILD and pathogenic TRG mutations, even though IPF was the most common diagnosis, all other ILDs, including IPAF, iPPFE, RA-ILD, HP, sarcoidosis, and pneumoconiosis were also seen [15,16]. However, despite differences in ILD patterns, TRG variants were characterised by relentlessly progressive disease in both IPF and non-IPF patients, suggesting that inherited telomere-related mutations are more predictive of accelerated progression than the underlying ILD pattern [15].

## 7. Common Telomere Related Gene SNPs and ILDs

Common single nucleotide polymorphisms (SNPs) are genetic variations that occur in >1% of the general population, often with an unknown effect on protein function or expression, and neither sufficient nor necessary to cause disease. In a two-stage genome wide association study (GWAS) of Japanese IPF patients, a significant association with a SNP in *TERT* (rs2736100) was identified [65]. This SNP has also been associated with ILD in patients of European descent, although when separated into IPF and other ILDs, the SNP was associated with other ILDs but not IPF, however the sample sizes were relatively small (IPF *n* = 84, other ILDs *n* = 143) [66]. A large GWAS of IIP patients of European descent (IIP *n* = 1616, controls *n* = 4683) found significant associations with rs2736100 and rs2853676 in *TERT* and rs1881984 near *TERC* [67]. *TERT* rs273600 was also borderline associated with disease in Mexican IPF patients, but not in Korean patients [68].

The associations between TRG SNPs and ILDs discussed in this section are summarised in Table 2.

## 8. Smoking, Telomeres, and ILDs

Smoking is the primary cause of respiratory bronchiolitis-ILD (RB-ILD), desquamative interstitial pneumonia and pulmonary Langerhans cell histiocytosis, and is a risk factor for the development of IPF and RA-ILD, although other ILDs including HP and sarcoidosis are less prevalent in smokers than non-smokers [69]. TL is shorter in ever smokers compared to never smokers, and in current smokers compared to never and former smokers, with an inverse relationship between pack-years and telomere length [70]. The average age of death in ILD smokers with *TERT* or *TERC* mutations is 10 years earlier than in mutation-carrying non-smokers [35], and an association between smoking history and pulmonary fibrosis in *TERT* mutation carriers ≥40 years of age has been shown [14]. In IPF, a disease characterised by a UIP pattern predominantly seen in older, male, ex-smokers, smoking status was not associated with TL. However, in the full cohort, including IPF and non-IPF patients, smoking status was associated with TL, but with a modest effect, estimated at only 70 bp, compared to IPF disease status [59].

## 9. Extra-Pulmonary Manifestations

There are a number of extra-pulmonary manifestations that can suggest a diagnosis of telomeropathy in a patient with pulmonary fibrosis [71]. These include premature hair greying, nail dystrophy, haematological abnormalities including leukopenia and thrombocytopenia, increased cancer risk, and liver function abnormalities/liver cirrhosis [72]. Mild haematological abnormalities, such as mild anemia or mildly reduced platelet counts have been described in patients with PF and TL < 25th centile, and could therefore raise a suspicion of a telomeropathy and suggest the need for more detailed testing [40].

## 10. Genetic Screening for Telomeropathy in Clinical Practice

Genetic testing for telomere variants is considered in patients with a family history of ILD (at least one family member), in patients with early onset IPF (<50 years), and in those in whom extrapulmonary features suggest a possible telomeropathy, as in the section above. A family history of pulmonary fibrosis can also be an expression of other gene mutations, such as surfactant-related genes, Nogee et al. reported the first recognised case of familial pulmonary fibrosis in 2001 [73], linked to a heterozygous mutation in surfactant protein C, with subsequent discovery of different mutations in the same gene and in other surfactant related genes, with substantial heterogeneity in clinical phenotypes. Familial pulmonary fibrosis can also occur in other genetically determined rare systemic disorders, such as Hermansky-Pudlak syndrome, an autosomal recessive disorder linked the mutations in *HPS* genes [3]. Algorithms to guide genetic testing decisions in patients with lung fibrosis have been suggested [71,74], and there is at least one published experience of the feasibility of a multidisciplinary team dedicated to inherited pulmonary fibrosis [75]. However, at the moment, only a limited number of specialist ILD centres perform genetic screening. Furthermore, there are currently no guidelines on genetic screening/standardised gene panels for TRG mutations/TL in patients with fibrotic ILD and their families. A European Respiratory Society (ERS) taskforce is currently working on providing an ERS statement on the indications for genetic testing in pulmonary fibrosis.

## 11. Treatment of ILDs and Telomeres

In recent years, anti-fibrotic treatment (nintedanib and pirfenidone) has been approved for use in IPF. In a retrospective analysis of 33 patients with fibrotic ILD (31 with IPF) carrying mutations in *TERT* or *TERC* and treated with pirfenidone, there was no reduction in the rate of FVC and DLCO decline compared to the rate before treatment, although treatment safety was similar to that seen in patients with sporadic IPF [76]. However, in a post hoc analysis of the pirfenidone trials CAPACITY and ASCEND, a significant treatment benefit of pirfenidone compared to placebo was observed in both subjects with TL above and below the median, although patients with TRG mutations (*n* = 102) had a more rapid FVC decline than non-carriers [59]. Similarly, in a recent retrospective review of a European cohort of 89 patients with IPF and TRG mutations, both pirfenidone and nintedanib were associated with a reduced rate of FVC decline compared to decline before treatment, with a similar side effect profile to that observed in the general IPF population [77]. These larger studies support the notion that anti-fibrotic treatment is of benefit in progressive fibrosis regardless of TL.

Telomeropathies may adversely affect the response to immunosuppression, by increasing the risk of bone marrow suppression and infections. The PANTHER trial was a landmark study demonstrating that immunosuppression was associated with increased lower respiratory tract infections, hospitalisations, and mortality in patients with IPF. Interestingly, a post hoc analysis of this and other IPF trial cohorts revealed that the risk of poor outcomes when exposed to immunosuppression was observed only in patients with leukocyte TL < 10th centile [78]. Interestingly, a link between short TL and poor response to immunosuppression has also been observed in fibrotic HP. Adegunsoye A. et al. reported that in patients with fibrotic HP with the lowest TL quartile, mycophenolate treatment was not associated with improved survival or lung function, in contrast to those with higher TL, in whom mycophenolate was associated with benefit. Whether the lack of benefit in the fibrotic HP patents with short TL was due to bone marrow toxicity or other adverse effects is not known [79].

Further evidence with regard to greater toxicity of immunosuppression comes from transplanted patients. Data suggest that patients with TRG mutations may suffer more complications following lung transplant, including haematological complications [80,81], anaemia post-transplant, leukopenia prompting change in immunosuppressive regimen, recurrent lower respiratory tract infections [82], restrictive chronic lung allograft dysfunction, and renal disease [83]. There is also some evidence that pulmonary fibrosis patients with TRG mutations have a higher risk of death following transplantation [83], although Borie et al. found no difference in median survival between patients with (*n* = 9) and without (*n* = 196) known mutations [81]. In 82 pulmonary fibrosis patients who underwent a lung transplant, those with TL < 10th percentile had worse survival (HR = 10.9) and shorter time to onset of chronic lung allograft dysfunction, as well as higher rates of allograft dysfunction [84], although another study found no association between donor or recipient lung telomere length and survival [85].

## 12. Telomere-Targeted Treatment

Therapeutic targeting of telomerase could represent a potential treatment option for telomeropathy-related pulmonary fibrosis. It has been shown that hormones and growth factors are involved in regulating telomerase activity and gene expression of *TERT*; there is an imperfect oestrogen response element within the *TERT* promoter, and oestrogen rapidly up-regulates *TERT* gene expression and telomerase activity [86]. Out of 16 patients with DC treated with androgen therapy, 69% had a haematological response [87]. Danazol is a synthetic androgen derivative. Two DC patients with heterozygous *TERC* mutations and bone marrow failure, treated with danazol, showed a good haematological response [88]. A case report of a female patient with premature greying, finger clubbing, hepatosplenomegaly, NSIP with respiratory failure (FVC 54%, DLCO 21%) and peripheral blood mononuclear cell (PBMC) TL < 1st centile, who was treated with prednisolone, mycophenolate mofetil and danazol, demonstrated dramatic improvement, with return to near normal lung function and improved radiological appearance, and TL within normal range [89]. A study of danazol attenuation of accelerated leukocyte telomere attrition in patients with telomeropathies was halted early because all 12 patients evaluated showed reduced TL attrition, with 92% showing a gain in leukocyte TL after 24 months. Pulmonary fibrosis scores based on CT were stable during the study in all but one patient, who died of acute exacerbation. Lung function tests prior to treatment were available for seven patients, all of whom had shown previous decline, but no significant decrease in DLCO [47] or FVC [90] during the study period.

A novel small molecule activator of telomerase, GRN510, given to bleomycin treated *Tert* heterozygous mice, activated telomerase activity 2–4-fold in hematopoietic progenitors ex vivo, and in bone marrow and lung tissue in vivo. GRN510 also suppressed the development of fibrosis in bleomycin treated mice with a reduction in inflammatory infiltrate and collagen deposition. No effect was seen when mice were also treated with a telomerase inhibitor, demonstrating that the effect of GRN510 is via activation of telomerase. Small airway epithelial cells and lung fibroblasts were also treated ex vivo, telomere activity was up-regulated only in the small airway epithelial cells, with decreased markers of senescence [91].

Another potential therapeutic avenue is inhibition of PAP-associated domain-containing protein 5 (PAPD5), which works in opposition to PARN, by targeting TERC RNA for degradation. Knockdown of PAPD5 boosts TERC stability, increases telomerase activity, and causes telomere lengthening in cells [21]. Two inhibitors of PAPD5, with potential therapeutic capacity, have been identified. Treatment of *PARN* mutant-induced pluripotent stem cells (iPSCs) from DC patients with the small-molecule PAPD5 inhibitor BCH001, reversed TERC 3′ end processing defects and resulted in elongation of telomere ends. In patient iPSCs carrying pathogenic *DKC1* mutations, treatment with BCH001 also enhanced TERC levels and maturation, telomerase activity, and telomere length [92]. Treatment with a novel PAPD5/7 inhibitor, RG7834, rescues TERC levels in *DKC1* and *PARN* knockdown HeLa cells. As a physiological model of DC, *DKC1* mutant human embryonic stem cells (hESCs) were generated. When treated with RG7834, TERC levels and teleromerase activity was increased [93].

## 13. Conclusions

Telomere shortening is associated with the aging process. For most fibrotic ILDs, the risk of disease markedly rises with increasing age. Shorter leukocyte TL is seen in a proportion of patients with both familial and sporadic ILD. Genetic variants in TRGs, both rare, functional mutations and more common polymorphisms of unknown effect, are associated with TL shortening and with incidence of ILD. Determination of TL and TRG testing may be a useful indicator of disease risk in asymptomatic relatives of familial ILD patients and of disease progression and treatment response in ILD patients. The relationship between ILD and telomere biology may suggest future routes to monitoring of telomeropathy-associated ILDs and, importantly, for treatment. Further studies on the relationship between TL, telomere-related gene mutations/variants and disease behaviour across the spectrum of fibrotic ILDs are needed. As knowledge of the links between telomeropathy and lung fibrosis increases, specific recommendations should be developed for genetic testing in patients with fibrotic lung disease and their family members. Recommendations are also needed to guide immunosuppressive treatment decisions and assessment of lung transplant risk and post-transplant treatment for patients with suspected telomeropathy.

## Figures and Tables

**Table 1:** Rare telomere related gene mutations and ILDs.

Disease	Gene	Reference
Familial IPF	*TERT*, *TERC*, *DKC1*, *TINF2*, *RTEL1*, *PARN*	Armanios et al. [18], Cronkhite et al. [13], Diaz de Leon et al. [14], Stuart et al. [51], Kannengiesser et al. [52], Cogan et al. [53], Kropski et al. [54], Kropski et al. [55], Alder et al. [56], Borie et al. [60]
Sporadic IPF	*TERT*, *TERC*, *RTEL1*, *PARN*	Cronkhite et al. [13], Diaz de Leon et al. [14], Dressen et al. [59], Petrovski et al. [61], Newton et al. [15], Borie et al. [16]
NSIP	*TERT*	Newton et al. [15]
DIP	*TERT*	Newton et al. [15]
PPFE	*TERT*, *TERC*, *RTEL1*	Newton et al. [15], Nunes et al. [64]
Unclassifiable ILD	*TERT*, *TERC*, *RTEL1*, *PARN*	Newton et al. [15]
CHP	*TERT*, *TERC*, *RTEL1*, *PARN*, *DKC1*	Newton et al. [15], Borie et al. [16], Ley et al. [63]
CTD-ILD	*TERT*, *TERC*, *RTEL1*	Newton et al. [15]
IPAF	*TERT*, *RTEL1*, *PARN*	Newton et al. [15], Borie et al. [16]
RA-ILD	*TERT*, *RTEL1*, *PARN*	Borie et al. [16], Juge et al. [62]
Sarcoidosis	*RTEL1*	Borie et al. [16]
Pneumoconiosis	*RTEL1*	Borie et al. [16]

IPF: Idiopathic pulmonary fibrosis, NSIP: Nonspecific interstitial pneumonia, DIP: Desquamative interstitial pneumonia, PPFE: Pleuroparenchymal fibroelastosis, ILD: Interstitial lung disease, CHP: Chronic hypersensitivity pneumonitis, CTD-ILD: Connective tissue disease associated interstitial lung disease, IPAF: Interstitial pneumonia with autoimmune features, RA-ID: Rheumatoid arthritis associated interstitial lung disease.

**Table 2:** Common SNPs and ILDs.

Gene	SNP	Disease	OR (95% CI) and *p*-Value	Population	Cohort Size (Patients/Controls)	Reference
*TERT*	rs2736100	IPF	OR = 2.11 (1.61–2.78) *p* = 2.9 × 10^−8^	Japanese	242 ^‡^/1469	Mushiroda et al. [65]
		Non-IPF ILD	OR = 1.43 (1.11–1.85) *p* = 6.2 × 10^−3^	European	143/689	Wei et al. [66]
		IPF	OR = 1.08 (0.78–1.49) *p* > 0.05	European	84/689	Wei et al. [66]
		IIP	OR not given *p* = 1.7 × 10^−19^	European	2492 ^‡^/6573	Fingerlin et al. [67]
		IPF	OR = 0.5 *p* = 0.05	Mexican	83/111	Peljto et al. [68]
		IPF	OR = 0.57 *p* = 0.17	Korean	239/87	Peljto et al. [68]
	rs2853676	IIP	OR not given *p* = 3.3 × 10^−8^	European	2492 ^‡^/6573	Fingerlin et al. [67]
*TERC*	rs1881984	IIP	OR not given *p* = 4.5 × 10^−8^	European	2492 ^‡^/6573	Fingerlin et al. [67]

^‡^ Meta-analysis of a discovery and replication cohort. IPF: Idiopathic Pulmonary Fibrosis, ILD: Interstitial Lung Disease, IIP: Idiopathic Interstitial Fibrosis.

## Data Availability

No new data were created or analyzed in this study. Data sharing is not applicable to this article.
